# Discrepancy Between Fasting Flow-Mediated Dilation and Parameter of Lipids in Blood: A Randomized Exploratory Study of the Effect of Omega-3 Fatty Acid Ethyl Esters on Vascular Endothelial Function in Patients With Hyperlipidemia

**DOI:** 10.1007/s12325-020-01286-1

**Published:** 2020-03-21

**Authors:** Tamio Teramoto, Hirotaka Shibata, Yuki Suzaki, Shingo Matsui, Naoto Uemura, Hirofumi Tomiyama, Akira Yamashina

**Affiliations:** 1 Teikyo Academic Research Center, Teikyo University, Tokyo, Japan; 2 Faculty of Medicine, Oita University, Oita, Japan; 3 Takeda Pharmaceutical Company Limited, Tokyo, Japan; 4 Department of Cardiology, Tokyo Medical University, Tokyo, Japan

**Keywords:** Dyslipidemia, Flow-mediated dilation, Hyperlipidemia, Lotriga^®^, Omega-3 fatty acid ethyl esters, Triglyceridemia, Vascular endothelial function

## Abstract

**Introduction:**

Omega-3 fatty acid ethyl esters (omega-3), an eicosapentaenoic acid and docosahexaenoic acid preparation (Lotriga^®^, Takeda Pharmaceutical Company Limited), are approved in Japan to treat triglyceridemia. We investigated the effects of omega-3 on vascular endothelial function, measured by flow-mediated dilation (FMD).

**Methods:**

Patients with dyslipidemia receiving 3-hydroxy-3-methyl-glutaryl-coenzyme A (HMG-CoA) reductase inhibitors were randomized 1:1 to receive omega-3 at 2 g (QD) or 4 g (2 g BID) for 8 weeks. The primary end point was the change from baseline of fasting  %FMD in each treatment group. Secondary end points included the 4-h postprandial  %FMD and 4-h postprandial triglyceride (TG) level.

**Results:**

Thirty-seven patients were randomized to receive omega-3 at 2 g (*n* = 18) or 4 g (*n* = 19). Mean fasting %FMD did not increase from baseline to week 8 in the 2-g group (− 1.2%) or 4-g group (− 1.3%). Mean 4-h postprandial %FMD did not change from baseline to week 8 in the 2-g group (0.0%), but increased in the 4-g group (1.0%). Mean 4-h postprandial TG level decreased by 34.7 mg/dl from baseline over week 8 in the 2-g group, with a significantly larger decrease in the 4-g group of 75.9 mg/dl (*p* < 0.001). No new safety concerns were identified.

**Conclusions:**

Fasting %FMD did not improve after 8 weeks of omega-3 treatment at 2 g or 4 g. After 8 weeks, 4-h postprandial TG levels showed improvement at both doses, with a greater reduction in the 4-g group.

**Trial Registration:**

ClinicalTrials.gov, ID: NCT02824432.

**Electronic supplementary material:**

The online version of this article (10.1007/s12325-020-01286-1) contains supplementary material, which is available to authorized users.

## Key Summary Points

**Table:** 

**Why carry out this study?**
Omega-3 fatty acid ethyl esters (omega-3) are approved in Japan for the treatment of triglyceridemia and have been shown to improve (i.e., increase) flow-mediated dilation (FMD); however, the correlation between changes in FMD and triglyceride (TG) or other lipid levels remains unknown.
To investigate the effects of omega-3 (2 g [*n* = 18]; 4 g [*n* = 19]) on vascular endothelial function, measured by FMD in patients with dyslipidemia receiving 3-hydroxy-3-methyl-glutaryl-coenzyme A reductase inhibitors, as measured by fasting %FMD, 4-h postprandial %FMD, fasting TG level, 4-h postprandial TG level and fasting plasma fatty acid levels.
**What was learned from this study?**
After 8 weeks of treatment, fasting %FMD did not improve in either group; however, improvements in 4-h postprandial %FMD, TG, total cholesterol, low-density lipoprotein-C and apolipoprotein B-48 were noted, moreso with omega-3 at 4 g; omega-3 was well tolerated at both doses, and no new safety concerns were identified.
Despite limitations, these study findings may help identify supplemental markers in addition to %FMD and support the development of non-invasive tests for atherosclerosis.

## Introduction

Dyslipidemia can be classified by elevated levels of low-density lipoprotein-cholesterol (LDL-C) and high triglyceride (TG) levels and decreased levels of high-density lipoprotein-cholesterol (HDL-C) [1, 2], and is a major risk factor for cardiovascular events [3]. 3-Hydroxy-3-methyl-glutaryl-coenzyme A (HMG-CoA) reductase inhibitors lower LDL-C and reduce the incidence of coronary deaths, coronary events [4–6] and the risk of other major vascular events such as strokes and coronary revascularizations [7]. However, HMG-CoA reductase inhibitors do not reduce TG levels to the same extent as LDL-C levels [6, 8].

The role of TG as a risk factor for cardiovascular disease has been strongly debated for a long time [9]. Epidemiologic studies suggest that elevated TG levels are associated with an increased risk of cardiovascular disease in patients with diabetes [10]. Elevated TG levels in the non-fasting state are associated with an increased risk of myocardial infarction, ischemic heart disease and death [11], while TG levels under fasting conditions are not considered to be associated with cardiovascular events [12]. In addition, insulin resistance associated with obesity and diabetes elevates TG and other lipid levels, the combination of which are considered to increase the risk of cardiovascular disease [13]. Furthermore, Mendelian randomization studies suggest that hypertriglyceridemia causally influences risk for coronary artery disease [14].

Omega-3 fatty acid ethyl esters (omega-3), an eicosapentaenoic acid (EPA) and docosahexaenoic acid (DHA) preparation (Lotriga^®^, Takeda Pharmaceutical Co., Ltd.), are approved and marketed in Japan for the treatment of triglyceridemia [15, 16]. Omega-3 significantly reduces fasting TG levels (*p* < 0.001), reduces TG levels in a dose-dependent manner, reduces LDL-C and interleukin-6 levels [17, 18] and is expected to reduce remnant-like particles (RLPs) [19]. In addition, the randomized phase 4 Japan EPA Lipid Intervention Study (JELIS) investigated the use of EPA plus a HMG-CoA reductase inhibitor in the treatment of patients with concurrent hypertriglyceridemia [20]. Results showed a reduction in TG levels and major coronary events with EPA plus HMG-CoA reductase inhibitor treatment, compared with a HMG-CoA reductase inhibitor alone [20].

Omega-3 polyunsaturated fatty acids have also been shown to also improve (i.e., increase) flow-mediated dilation (FMD) [17, 21], which is a noninvasive measure of blood vessel health [22] that has been utilized in studies in Japan [23, 24]. To our knowledge, there are few reports of FMD under fasting and non-fasting conditions. However, a time-course analysis of FMD in African American participants found that baseline brachial artery diameter significantly (*p* = 0.03) increased 2 h after a high-fat meal, indicating the importance of measurements under postprandial conditions [25].

Despite the above findings, the mechanism of action and dose dependency of omega-3 on FMD, TG levels and other lipid levels under fasting and postprandial conditions in patients with hyperlipidemia have not been fully elucidated [26]. We therefore investigated the effects of omega-3 at 2-g and 4-g doses on vascular endothelial function (as measured by FMD), TG levels and other lipid levels under fasting and postprandial conditions in patients with concurrent hypertriglyceridemia receiving HMG-CoA reductase inhibitors.

## Methods

This was a multicenter, open-label, randomized study investigating the effects of omega-3 on vascular endothelial function, measured by FMD, in patients with concurrent hypertriglyceridemia (TG ≥ 150 mg/dl [27]) who were receiving an HMG-CoA reductase inhibitor (NCT02824432). The study design is shown in Supplementary Fig. S1. Written informed consent was obtained from each patient included in the study at visit 1 (day − 29 to − 1). Eligibility was screened and baseline measurements obtained at visit 2 (day − 15 to − 1). Patients started taking the study drug the day after visit 2. Patients were randomized 1:1 to receive oral granular capsules of omega-3-acid ethyl esters at 2 g (QD) or 4 g (2 g BID) immediately after their meal for up to 8 weeks.

Randomization was stratified based on fasting TG levels to mitigate the potential impact of factors that affect FMD between treatment groups. Information on randomization was only available to authorized persons. The principal investigator or investigator(s) prescribed the study drug (2 g or 4 g) as notified by the case registration web system and recorded the drug information for each patient into the case report form. Measurements after 4 weeks of treatment were obtained at visit 3 (day 14 to 41) and after 8 weeks at visit 4 (day 42 to 70). As some patients had to visit a testing laboratory for their FMD measurement in addition to the study site where they received treatment, a total of three visits were added. The number of visits during the treatment period was a minimum of three and a maximum of six. This study was conducted in seven medical institutions in Japan. All procedures performed in studies involving human participants were in accordance with the ethical standards of the institutional and/or national research committee and with the 1964 Helsinki Declaration and its later amendments or comparable ethical standards. Informed consent was obtained from all individual participants included in the study. The study protocol, informed consent form and other regulation-specified documents were reviewed and approved by the independent ethics committee of each site.

Eligible patients were men or postmenopausal women aged ≥ 20 years at the time of informed consent at visit 1, diagnosed with dyslipidemia and receiving instructions for lifestyle improvement, as well as continuous treatment with a stable dose of an HMG-CoA reductase inhibitor for at least 4 weeks before informed consent. All patients were required to have a fasting TG level of 150–499 mg/dl at visit 1. Patients were excluded if they had: a history of cardiovascular disease, including revascularization or coronary artery disease (not stroke); an aortic aneurysmectomy within 24 weeks prior to visit 1; any clinically significant hemorrhagic disorders; a fasting  %FMD level of 0% at start of study drug administration at visit 2; had received antidyslipidemic agents within 4 weeks prior to informed consent; or had experienced a change in dose of dyslipidemia agents, antidiabetic or antihypertensive agents between informed consent at visit 1 and study drug administration at visit 2.

The primary end point was the change from baseline of fasting  %FMD in each treatment group. Secondary end points were 4-h postprandial  %FMD, fasting TG level, 4-h postprandial TG level and fasting plasma fatty acid fraction levels [dihomo-gamma-linolenic acid, arachidonic acid (AA), EPA, DHA and EPA/AA ratio]. Additional efficacy end points were total cholesterol, LDL-C, HDL-C, RLP-cholesterol (RLP-C), apolipoprotein B-48, C-reactive protein (CRP) and urinary 8-epi prostaglandin F2α (8-epi-PGF2α). Adverse events (AEs) were a safety end point.

Fasting  %FMD was measured on visit 2 (day − 15 to − 1), visit 3 (day 14 to 41), visit 4 (day 42 to 70) and up to 3 days after the last dose. Four-hour postprandial  %FMD was measured on visit 2 (day − 15 to − 1), visit 4 (day 42 to 70) and up to 3 days after the last dose. The fasting test was performed under ≥ 10-h fasting conditions, and the 4-h postprandial test was performed 4 h after consumption of a high-fat meal (allowable range ± 30 min).  %FMD was measured by physicians or clinical laboratory technicians in a dedicated room in the morning using a device equipped with software to monitor the brachial artery diameter (UNEXEF 38G; UNEX Co. Ltd., Nagoya, Japan). Physicians and technicians completed a short training course on the theory of FMD, test equipment, anatomical knowledge of the blood vessels to be measured, measurement techniques and handling when the probe was displaced; they then completed approximately 1 month of voluntary training before measuring %FMD in patients. A pneumatic cuff was placed on the forearm, and pressure was applied for 5 min. %FMD was measured by comparing the pre-vascularization value with the maximum vascular diameter of the brachial artery after the perfusion. Nitrates were not used.

In this study, an additional and exploratory evaluation of biomarkers for  %FMD was performed in the Integrated Technology Research Laboratories (Takeda Pharmaceutical Company Limited, Tokyo, Japan). A comprehensive evaluation was conducted for blood proteins, unsaturated fatty acid metabolites and lipids. All samples were collected under fasting conditions at visit 1 (day − 29 to − 1), visit 3 and visit 4; additionally, 4-h postprandial lipid samples were also collected at visit 1 and visit 4 (Supplementary Table S4).

The planned sample size was based on the feasibility to explore the effects of omega-3 on vascular endothelial function. The full analysis set (FAS) and safety analysis set (SAS) were both defined as patients who were randomized and received at least one dose of the study drug. For all end points, descriptive statistics [number of patients, mean and standard deviation (SD)] and a two-sided 95% confidence interval (CI) for the mean were calculated for each treatment group at each time point, and the change in mean ± SD was calculated at week 4 and week 8 (visit 3 or visit 4 minus visit 2). For comparisons before and after administration at each time point (at week 0 vs. week 4 or week 8), a corresponding *t* test was performed for each group, and *p* values were calculated. Least squares (LS) mean percentage change was compared between treatment groups using an analysis of covariance model adjusted by baseline fasting TG (at week 0). For biomarker evaluation, at each time point of the treatment period, the amount of change from week 0 for each item was calculated and the coefficient of correlation with the amount of change of  %FMD was calculated at 8 weeks. Correlation coefficients were calculated with the change of  %FMD for each treatment group on a scatter plot. Statistical analyses were performed using SAS (version 9.4, The SAS institute, Cary, NC).

AEs were coded using the Medical Dictionary for Regulatory Activities (MedDRA) version 20.0 and classified by System Organ Class and Preferred Term.

## Results

### Patient Disposition and Baseline Characteristics

From August 2016 to August 2017, 37 patients were randomized to receive omega-3-acid ethyl esters 2 g (2-g group; *n* = 18) or 4 g (4-g group; *n* = 19). One patient in the 2-g group did not complete the study because of a major deviation of protocol entry criteria. Patient disposition is shown in Supplementary Fig. S2.

Demographic and other baseline characteristics were similar between the two treatment groups and representative of the population under study (Table 1), albeit with some exceptions between treatment groups. A larger proportion of patients in the 2-g group than in the 4-g group consumed fish approximately every 2 days (27.8% vs. 10.5%, respectively), had never smoked (72.2% vs. 47.4%, respectively) or had 4-h postprandial TG level (treatment period) < 200 mg/dl (33.3% vs. 5.3%, respectively). A smaller proportion of patients in the 2-g group than the 4-g group rarely consumed fish (5.6% vs. 21.1%, respectively), were ex-smokers (27.8% vs. 52.6%, respectively) or had 4-h postprandial TG level ≥ 200 to < 500 mg/dl (66.7% vs. 94.7%, respectively). The postmenopausal period was shorter in the 2-g group (9.9 years) than in the 4-g group (16.6 years).

**Table 1:** Demographic and baseline characteristics (SAS)

	Omega-3	
	2 g	4 g
	*n* = 18	*n* = 19
Mean age, years (SD)	58.5 (11.1)	61.5 (7.9)
Age categories, *n* (%)		
< 65 years	11 (61.1)	11 (57.9)
≥ 65 to < 75 years	6 (33.3)	7 (36.8)
≥ 75 years	1 (5.6)	1 (5.3)
Gender, *n* (%)		
Male	7 (38.9)	8 (42.1)
Female	11 (61.1)	11 (57.9)
Mean height, cm (SD)	158.4 (9.2)	162.1 (9.2)
Mean weight, kg (SD)	68.3 (13.3)	71.9 (14.6)
Mean BMI, kg/m^2^ (SD)	27.1 (4.1)	27.1 (3.5)
BMI categories, *n* (%)		
≥ 18.5 to < 25.0 kg/m^2^	5 (27.8)	7 (36.8)
≥ 25.0 kg/m^2^	13 (72.2)	12 (63.2)
Mean duration of dyslipidemia, years (SD)	9.2 (5.9)	9.5 (5.2)
Frequency of consumption of fish, *n* (%)		
Almost every day	1 (5.6)	1 (5.3)
About every 2 days	5 (27.8)	2 (10.5)
About once or twice per week	11 (61.1)	12 (63.2)
Rarely	1 (5.6)	4 (21.1)
Smoking history, *n* (%)		
Never smoked	13 (72.2)	9 (47.4)
Current smoker	0 (0.0)	0 (0.0)
Ex-smoker	5 (27.8)	10 (52.6)
History of drinking, *n* (%)		
Yes	4 (22.2)	2 (10.5)
No	14 (77.8)	17 (89.5)
Mean fasting TG level, mg/dl (SD)^a^	176.8 (59.2)	194.4 (48.6)
Fasting TG levels, *n* (%)^a^		
< 200 mg/dl	13 (72.2)	12 (63.2)
≥ 200 to < 500 mg/dl	5 (27.8)	7 (36.8)
Mean 4-h postprandial TG level, mg/dl (SD)^a^	265.9 (102.6)	278.2 (70.5)
4-h postprandial TG levels, *n* (%)^a^		
< 200 mg/dl	6 (33.3)	1 (5.3)
≥ 200 to < 500 mg/dl	12 (66.7)	18 (94.7)
Mean EPA/AA ratio (SD)^a^	0.27 (0.116)	0.24 (0.155)
Postmenopausal period, years	*n* = 9	*n* = 8
Mean (SD)	9.9 (6.1)	16.6 (5.9)

*AA* arachidonic acid, *BMI* body mass index, *EPA* eicosapentaenoic acid, *Omega*-*3* omega-3 fatty acid ethyl esters, *SAS* safety analysis set, *SD* standard deviation, *TG* triglyceride

^a^Measurement taken at visit 2

### Efficacy

**Fig. 1:**
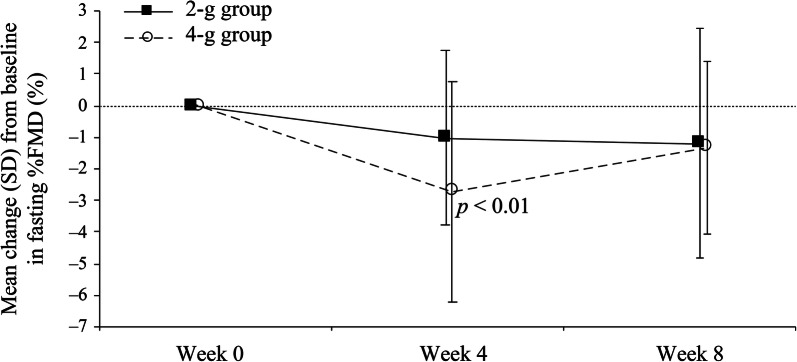
Means plots of change from baseline in fasting %FMD by visit in the omega-3 2-g group and 4-g group. *FMD* flow-mediated dilation, *Omega*-*3* omega-3 fatty acid ethyl esters, *SD* standard deviation

Summary statistics for fasting %FMD by treatment group are shown in Table 2. No increase in mean fasting %FMD was observed from baseline to week 8 in both the 2-g group (− 1.2 ± 3.64%) and 4-g group (− 1.3 ± 2.75%) (Fig. 1).

**Table 2:** Summary statistics for fasting %FMD for the omega-3 2-g group and 4-g group (FAS)

Analysis visit	Statistics	Observation value		Change from baseline	
		2-g group	4-g group	2-g group	4-g group
Fasting %FMD					
Week 0	*n*	17	17		
	Mean (SD)	6.7 (3.47)	5.9 (3.62)		
	95% CI	4.92, 8.49	3.99, 7.71		
Week 4	*n*	16	17	15	17
	Mean (SD)	5.9 (1.92)	3.2 (2.90)	− 1.0 (2.78)	− 2.71 (3.48)
	95% CI	4.83, 6.87	1.66, 4.64	− 2.56, 0.52	− 4.50, − 0.92
	*p* value vs. week 0			0.178	*p *< 0.01
Week 8	*n*	16	17	15	15
	Mean (SD)	5.4 (2.28)	4.0 (2.24)	− 1.2 (3.64)	− 1.3 (2.75)
	95% CI	4.17, 6.60	2.79, 5.10	− 3.21, 0.81	− 2.86, 0.19
	*p* value vs. week 0			0.222	0.082
	*p* value 2 g vs. 4 g			0.212	

*CI* confidence interval, *FAS* full analysis set, *FMD* flow-mediated dilation, *Omega*-*3* omega-3 fatty acid ethyl esters, *SD* standard deviation

**Fig. 2:**
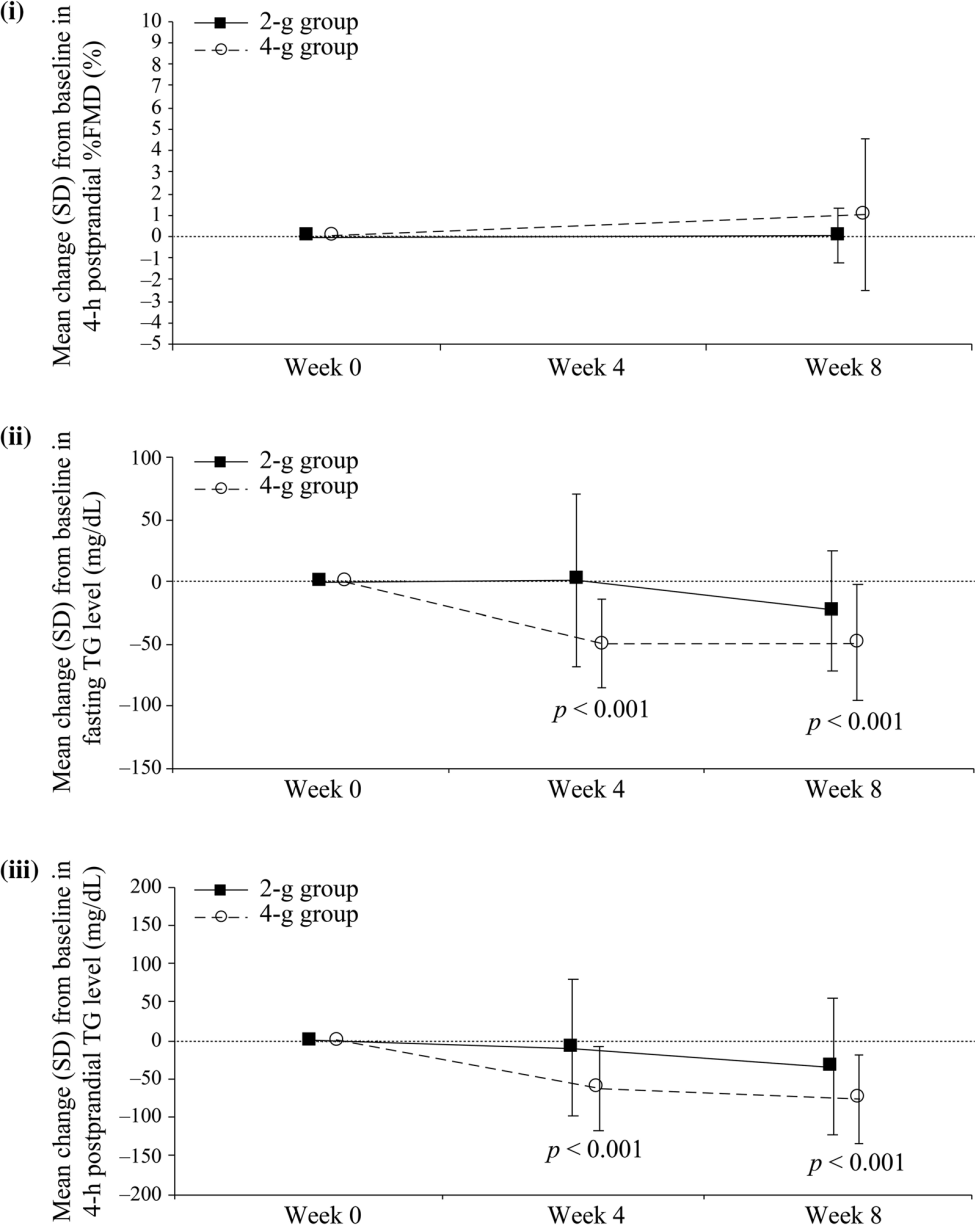
Means plots of change from baseline in (i) 4-h postprandial  %FMD, (ii) fasting TG level and (iii) 4-h postprandial TG level by visit in the omega-3 2-g group and 4-g group. *FMD* flow-mediated dilation, *h* hour, *Omega*-*3* omega-3 fatty acid ethyl esters, *SD* standard deviation, *TG* triglyceride

Summary statistics for 4-h postprandial %FMD, fasting TG level and 4-h postprandial TG level by treatment group are shown in Table 3. There was no significant increase in mean 4-h postprandial  %FMD from baseline to week 8 (Fig. 2i). Mean change (i.e., the degree of decrease) of 4-h postprandial %FMD from fasting %FMD in each group is shown in Supplementary Fig. S3. At baseline, the observed value of 4-h postprandial %FMD minus fasting  %FMD was − 1.4 ± 3.52% in the 2-g group and − 1.6 ± 3.52% in the 4-g group. However, 4-h postprandial %FMD minus fasting %FMD at week 8 showed a slight increase in both groups (0.4 ± 2.16% and 0.8 ± 2.82% in the 2-g and 4-g groups, respectively). Over the 8-week study period from baseline, both mean fasting TG level and mean 4-h postprandial TG level significantly decreased in the 4-g group only (both *p* < 0.001) (Fig. 2ii, iii).

**Table 3:** Summary statistics for 4-h postprandial  %FMD, fasting TG level and 4-h postprandial TG level for the omega-3 2-g group and 4-g group (FAS)

Analysis visit	Statistics	Observation value		Change from baseline	
		2-g group	4-g group	2-g group	4-g group
4-h postprandial %FMD^a^					
Week 0	*n*	14	15		
	Mean (SD)	5.5 (2.49)	3.8 (2.27)		
	95% CI	4.05, 6.92	2.54, 5.06		
Week 8	*n*	15^b^	18^b^	13	15
	Mean (SD)	5.8 (2.85)	5.0 (2.52)	0.0 (1.28)	1.0 (3.52)
	95% CI	4.24, 7.39	3.70, 6.20	− 0.76, 0.79	− 0.93, 2.97
	*p* value vs. week 0			0.966	0.280
	*p* value 2 g vs. 4 g			0.806	
Fasting TG level (mg/dl)					
Week 0	*n*	18	19		
	Mean (SD)	176.8 (59.2)	194.4 (48.6)		
	95% CI	147.4, 206.3	171.0, 217.8		
Week 4	*n*	18	19	18	19
	Mean (SD)	178.2 (75.5)	144.6 (39.4)	1.4 (69.6)	− 49.8 (35.7)
	95% CI	140.7, 215.8	125.6, 163.5	− 33.2, 36.0	− 67.0, − 32.6
	*p* value vs. week 0			0.933	*p *< 0.001
Week 8	*n*	17	19	17	19
	Mean (SD)	157.2 (45.8)	144.9 (43.1)	− 23.1 (48.4)	− 49.4 (46.7)
	95% CI	133.6, 180.7	124.2, 165.7	− 47.9, 1.8	− 71.9, − 26.9
	*p* value vs. week 0			0.067	*p *< 0.001
	*p* value 2 g vs. 4 g			0.038	
4-h postprandial TG level (mg/dl)					
Week 0	*n*	18	19		
	Mean (SD)	265.9 (102.6)	278.2 (70.5)		
	95% CI	214.9, 317.0	244.2, 312.2		
Week 4	*n*	17	19	17	19
	Mean (SD)	266.1 (75.2)	216.2 (79.1)	− 9.1 (89.3)	− 62.1 (54.3)
	95% CI	227.4, 304.7	178.0, 254.3	− 55.0, 36.8	− 88.2, − 35.9
	*p* value vs. week 0			0.679	*p *< 0.001
Week 8	*n*	17	19	17	19
	Mean (SD)	240.5 (74.7)	202.3 (78.3)	− 34.7 (89.4)	− 75.9 (57.0)
	95% CI	202.1, 278.9	164.5, 240.0	− 80.7, 11.3	− 103.4, − 48.5
	*p* value vs. week 0			0.129	*p *< 0.001
	*p* value 2 g vs. 4 g			0.322	

*CI* confidence interval, *FAS* full analysis set, *FMD* flow-mediated dilation, *h* hour, *Omega*-*3* omega-3 fatty acid ethyl esters, *SD* standard deviation, *TG* triglyceride

^a^Measured at week 0 and week 8 only

^b^Date of  %FMD measurement was available for more patients at week 8 than week 0

Summary statistics for fasting plasma fatty acid fraction levels are shown in Supplementary Table S1. Over the 8-week study period from baseline mean fasting dihomo-gamma-linolenic acid levels significantly decreased in the 2-g group and 4-g group (both *p *< 0.001). Mean fasting AA levels significantly decreased from baseline to week 8 in the 4-g group only (*p *< 0.001). For both plasma fatty acid types, there were statistically significant differences between the two groups at week 8 (Supplementary Table S1).

Summary statistics for additional efficacy end points are shown in Supplementary Table S2. Significant decreases from baseline to week 8 were only observed in the 4-g group for mean fasting total cholesterol (*p *< 0.001), mean 4-h postprandial total cholesterol (*p *< 0.001), mean fasting LDL-C (*p *< 0.001) and mean 4-h postprandial LDL-C (*p *< 0.01). Significant decreases in mean fasting RLP-C levels and mean fasting apolipoprotein B-48 levels were observed in the 4-g group only (*p *< 0.001 and *p* = 0.002, respectively). Mean urinary 8-epi-PGF2α levels showed almost no change from baseline to week 8 in the 2-g and 4-g group.

Results of biomarker evaluation are shown in Supplementary Table S5i, ii, iii. Regarding the panel of blood proteins and unsaturated fatty acid metabolites examined, the coefficients of correlation with  %FMD are shown in only 12 blood proteins and 5 unsaturated fatty acid metabolites, since some were below the detection limit. As for the panel of lipids, since > 700 lipids were measured, the coefficient of correlations with  %FMD were calculated only for the lipids that were significantly changed in concentration from week 0. There were no notable changes in blood proteins and unsaturated fatty acid metabolites. In the panel of lipids, a correlation of coefficient ≥ 0.4 was observed in some lipids, including acylcarnitine, diacylglycerol, phosphatidylcholine and sphingomyelin. However, no dose-dependency was observed, and a correlation of coefficient ≥ 0.4 was not regularly observed.

### Safety

An overview of AEs is shown in Supplementary Table S6. Four (22.2%) patients in the 2-g group and two (10.5%) in the 4-g group experienced AEs. In the 2-g group, two (11.1%) patients experienced gastrointestinal disorders (constipation, *n* = 1; diarrhea, *n* = 1; vomiting, *n* = 1), and two (11.1%) experienced infections and infestations (upper respiratory tract infection, *n* = 1; viral upper respiratory tract infection, *n* = 1). In the 4-g group, one (5.3%) patient experienced pharyngitis and one (5.3%) experienced lumbar spinal stenosis.

All AEs were mild or moderate in intensity. One (5.6%) patient in the 2-g group had a drug-related AE, which was mild in intensity. No serious AEs or AEs leading to drug discontinuation were reported, and no patients died on study.

## Discussion

In this exploratory study of omega-3 in patients with hyperlipidemia receiving HMG-CoA reductase inhibitors, there was no improvement (measured as an increase) in fasting  %FMD after 8 weeks of treatment at either 2 g or 4 g. However, levels of 4-h postprandial  %FMD and TG, total cholesterol, LDL-C and apolipoprotein B-48 (measured as a decrease) indicated numerical and partially statistically significant improvement after treatment for 8 weeks, with a higher magnitude of improvement with a 4-g dose of omega-3. Omega-3 was well tolerated at 2-g and 4-g doses, and no new safety concerns were identified.

For the primary end point of change from baseline of fasting  %FMD in each treatment group, the mean baseline value was > 5.0% in both treatment groups with large SDs. As a  %FMD of approximately 6.0% may be applied as a reference value in Japanese men and women aged ≥ 50 years, endothelial dysfunction may occur at lower values of  %FMD (e.g., < 5.0%) [28]. There were no signs of endothelial dysfunction in patients with mean baseline FMD < 5.0% or > 5.0%; however, a higher mean baseline value of fasting  %FMD (> 5.0%) in both treatment groups may have been measured, which may have in turn impacted the apparent lack of improvement in fasting  %FMD at week 8. In contrast, 4-h postprandial  %FMD had a lower mean value at baseline in both treatment groups and improved, albeit not significantly, at week 8. Furthermore, the mean change in 4-h postprandial  %FMD from fasting  %FMD for each patient at week 8 increased, albeit not significantly, while postprandial  %FMD is generally known to be lower in value compared with fasting  %FMD. We therefore consider our results notable, as postprandial  %FMD did not decrease at week 8 of omega-3 treatment. However, data obtained from measurements of 4-h postprandial  %FMD may have less variation than those from fasting  %FMD as these data were obtained on the second measurement from the same patient on the same day, and most likely by the same operator. Furthermore, the more frequently the same operator measures the same patient, the more accurate the measurement of  %FMD may become. Therefore, the 4-h postprandial data obtained from the second measurement may also be more reliable than that from their first measurement under fasting conditions.

The decrease in fasting TG in patients with hyperlipidemia who received omega-3 in the present study is consistent with that reported in patients with metabolic syndrome who received omega-3, and that in fasting and postprandial TG is consistent with that reported in patients with hypercholesterolemia who received EPA [17, 20]. Further study is required to determine whether the decrease in 4-h postprandial TG correlates with the increase in 4-h postprandial  %FMD in the present study.

For additional efficacy end points, most of the lipid parameters, including RLP-C, showed an improvement (measured as a decrease) in lipid levels. Improvements in lipid levels tended to be larger in the 4-g group than in the 2-g group. In particular, the significant decrease in mean fasting apolipoprotein B-48 levels in the 4-g group is encouraging, given that fasting serum apolipoprotein B-48 levels are significantly correlated (*p* < 0.0001) with the prevalence of coronary artery disease [29]. We consider these results to support the efficacy of omega-3 for the treatment of dyslipidemia. Moreover, the minor changes in HDL-C value are consistent with other studies reporting that the levels of HDL-C in patients administered HMG-CoA reductase inhibitors are not affected by additional treatment with omega-3 [20, 30].

Postprandial hyperlipidemia is a residual risk factor, and postprandial hypertriglyceridemia is an established risk factor of cardiovascular disease [26, 31]. Compared with the fasting state, postprandial hyperlipidemia is related to increasing levels of chylomicron remnants, which include TG and apolipoprotein B-48 [32–34]. The RLP-C measured in the present study reflects whole remnant lipoproteins including the chylomicron remnants. In addition, mean 4-h postprandial RLP-C levels were dose-dependently and significantly decreased from baseline to week 8 in the 4-g group.

Recently, the randomized, placebo-controlled VITAL trial investigated the administration of omega-3 and vitamin D in the primary prevention of cardiovascular disease and cancer among men aged ≥ 50 and women aged ≥ 55 years from the general population in the USA [35]. However, supplementation with omega-3 did not result in a lower incidence of major cardiovascular events or cancer than placebo in these patients [35]. However, the multicenter, randomized, double-blind, placebo-controlled REDUCE-IT trial investigated the administration of 2 g of icosapent ethyl twice daily in patients with elevated triglyceride levels who were also receiving statin therapy [36]. Results showed that the risk of ischemic events, including cardiovascular death, was significantly lower among those who received 2 g of icosapent ethyl twice daily than among those who received placebo [36]. Further investigation of ischemic events and end points from our present study (e.g., 4-h postprandial TG levels) in patients with elevated triglyceride levels who are also receiving statins would be of interest.

The present study contains limitations, which may have contributed to the primary end point not being met, and in turn the observed discrepancy with blood lipids. First, the study is limited by the small number of patients randomized and relatively short treatment period. In addition, a control group was not included as this was an exploratory study to determine the feasibility of investigating the effects of omega-3 on vascular endothelial function by FMD. Furthermore, the dose of HMG-CoA reductase inhibitors was not controlled in this study. Randomized controlled studies with a larger number of patients and a longer treatment period may be required for an appropriate analysis of the effect of omega-3 on dyslipidemia in patients receiving long-term treatment with HMG-CoA reductase inhibitors. In addition, postprandial data were not included in the main analysis, and long-term follow-up measurements of %FMD were not taken. Furthermore, the accuracy of the data obtained with FMD is dependent on the skill of the operator [22, 37]. However, the reliability of semiautomatic FMD assessment (scan and analysis) conducted in individual institutions in Japan has been reported as acceptable when limited to clear recordings [24]. Although the frequent, non-invasive measurement of  %FMD at baseline may provide more accurate data, frequent measurement still places a burden on patients. Further study is required to identify a standardized measurement and definition of a normal range for  %FMD. Finally,  %FMD is also affected by room temperature, and fasting  %FMD was measured year round without a controlled room temperature. Clearly, the current study design is limited, and with the large number of parameters examined no clear message can be drawn. However, we hope the findings will help to identify supplementary markers to  %FMD in the future and to support the development of non-invasive tests for atherosclerosis.

## Conclusion

Fasting  %FMD did not improve in patients with hyperlipidemia treated with omega-3 for 8 weeks. However, 4-h postprandial  %FMD, TG, total cholesterol, LDL-C and apolipoprotein B-48 all indicated numerical and partially statistically significant improvement after treatment for 8 weeks, with a higher magnitude of improvement with a 4-g dose of omega-3. Further study is required to determine the mechanism of action of omega-3 with regard to vascular endothelial function.

## Electronic supplementary material

Below is the link to the electronic supplementary material.

Supplementary material 1 (DOCX 252 kb)

Supplementary material 2 (PDF 79 kb)
