# Supplementary material for: Discrepancy Between Fasting Flow-Mediated Dilation and Parameter of Lipids in Blood: A Randomized Exploratory Study of the Effect of Omega-3 Fatty Acid Ethyl Esters on Vascular Endothelial Function in Patients With Hyperlipidemia
Source: Adv Ther. 2020 Mar 21;37(5):2169–83. doi: 10.1007/s12325-020-01286-1 (PMC7467499; doi:10.1007/s12325-020-01286-1)
Supplement: Supplementary file 1 — Supplementary material 1 (DOCX 252 kb) [file 12325_2020_1286_MOESM1_ESM.docx]

Supplementary Material

**Supplementary Table S1** Summary statistics for fasting plasma fatty acid fraction levels for the Omega-3 2-g group and 4-g group (FAS)

| **Analysis visit** | **Statistics** | **Observation value** | | **Change from baseline** | |
| --- | --- | --- | --- | --- | --- |
|  |  | **2-g group** | **4-g group** | **2-g group** | **4-g group** |
| Fasting dihomo-gamma-linolenic acid (μg/mL) | | | | | |
| Week 0 | *n* | 18 | 19 |  |  |
|  | Mean (SD) | 51.4 (14.8) | 57.3 (12.3) |  |  |
| Week 8 | *n* | 17 | 19 | 17 | 19 |
|  | Mean (SD) | 43.1 (9.1) | 33.3 (8.6) | –9.9 (9.3) | –24.0 (10.1) |
|  | *p*-value *vs.* week 0 | | | *p <* 0.001 | *p <* 0.001 |
|  | *p*-value 2 g *vs.* 4 g | | | *p <* 0.001 | |
| Fasting AA (μg/mL) | | | | | |
| Week 0 | *n* | 18 | 19 |  |  |
|  | Mean (SD) | 254 (52.3) | 253 (70.0) |  |  |
| Week 8 | *n* | 17 | 19 | 17 | 19 |
|  | Mean (SD) | 243 (76.3) | 198 (50.0) | –11 (39.2) | –55 (36.6) |
|  | *p*-value *vs.* week 0 | | | 0.286 | *p <* 0.001 |
|  | *p*-value 2 g *vs.* 4 g | | | *p <* 0.001 | |
| Fasting EPA (μg/mL) | | | | | |
| Week 0 | *n* | 18 | 19 |  |  |
|  | Mean (SD) | 66 (29.2) | 56 (27.0) |  |  |
| Week 8 | *n* | 17 | 19 | 17 | 19 |
|  | Mean (SD) | 135 (41.2) | 194 (45.7) | 71 (32.2) | 138 (53.1) |
|  | *p*-value *vs.* week 0 | | | *p <* 0.001 | *p <* 0.001 |
|  | *p*-value 2 g *vs.* 4 g | | | *p <* 0.001 | |
| Fasting DHA (μg/mL) | | | | | |
| Week 0 | *n* | 18 | 19 |  |  |
|  | Mean (SD) | 173 (51.9) | 145 (45.0) |  |  |
| Week 8 | n | 17 | 19 | 17 | 19 |
|  | Mean (SD) | 201 (48.0) | 208 (48.7) | 26 (31.1) | 62 (51.7) |
|  | *p*-value *vs.* week 0 | | | *p <* 0.01 | *p <* 0.001 |
|  | *p*-value 2 g *vs.* 4 g | | | 0.075 | |
| Fasting EPA/AA ratio | | | | | |
| Week 0 | *n* | 18 | 19 |  |  |
|  | Mean (SD) | 0.27 (0.116) | 0.24 (0.155) |  |  |
| Week 8 | *n* | 17 | 19 | 17 | 19 |
|  | Mean (SD) | 0.58 (0.181) | 1.04 (0.388) | 0.32 (0.130) | 0.80 (0.324) |
|  | *p*-value *vs.* week 0 | | | *p <* 0.001 | *p <* 0.001 |
|  | *p*-value 2 g *vs.* 4 g | | | *p <* 0.001 | |
| Fasting DHA/AA ratio | | | | | |
| Week 0 | *n* | 18 | 19 |  |  |
|  | Mean (SD) | 0.70 (0.216) | 0.62 (0.280) |  |  |
| Week 8 | *n* | 17 | 19 | 17 | 19 |
|  | Mean (SD) | 0.88 (0.267) | 1.12 (0.418) | 0.18 (0.151) | 0.50 (0.300) |
|  | *p­*-value *vs.* week 0 | | | *p <* 0.001 | *p <* 0.001 |
|  | *p*-value 2 g *vs.* 4 g | | | *p <* 0.001 | |

*AA* arachidonic acid, *DHA* docosahexaenoic acid, *EPA* eicosapentaenoic acid, *FAS* full analysis set, *Omega-3* omega-3 fatty acid ethyl esters, *SD* standard deviation

**Supplementary Table S2** Summary statistics for additional efficacy endpoints for the Omega-3 2-g group and 4-g group (FAS)

| **Analysis visit** | **Statistics** | **Observation value** | | **Change from baseline** | |
| --- | --- | --- | --- | --- | --- |
|  |  | **2-g group** | **4-g group** | **2-g group** | **4-g group** |
| Total fasting cholesterol (mg/dL) | | | | | |
| Week 0 | *n* | 18 | 19 |  |  |
|  | Mean (SD) | 191 (34.1) | 188 (21.4) |  |  |
| Week 8 | *n* | 17 | 19 | 17 | 19 |
|  | Mean (SD) | 194 (45.9) | 174 (25.9) | 0 (27.4) | –14 (14.8) |
|  | *p*-value *vs.* week 0 | | | 0.972 | *p* < 0.001 |
|  | *p*-value 2 g *vs.* 4 g | | | 0.088 | |
| 4-h postprandial total cholesterol (mg/dL) | | | | | |
| Week 0 | *n* | 18 | 19 |  |  |
|  | Mean (SD) | 194 (33.2) | 187 (19.5) |  |  |
| Week 8 | *n* | 17 | 19 | 17 | 19 |
|  | Mean (SD) | 197 (45.6) | 172 (26.7) | –1 (27.1) | –15 (15.7) |
|  | *p*-value *vs.* week 0 | | | 0.937 | *p* < 0.001 |
|  | *p*-value 2 g *vs.* 4 g | | | 0.079 | |
| Fasting LDL-C (mg/dL) | | | | | |
| Week 0 | *n* | 18 | 19 |  |  |
|  | Mean (SD) | 117 (31.2) | 111 (16.7) |  |  |
| Week 8 | *n* | 17 | 19 | 17 | 19 |
|  | Mean (SD) | 119 (39.3) | 100 (21.1) | 0 (22.5) | –12 (11.6) |
|  | *p*-value *vs.* week 0 | | | 0.949 | *p* < 0.001 |
|  | *p*-value 2 g *vs.* 4 g | | | 0.116 | |
| 4-h postprandial LDL-C (mg/dL) | | | | | |
| Week 0 | *n* | 18 | 19 |  |  |
|  | Mean (SD) | 115 (29.0) | 107 (16.7) |  |  |
| Week 8 | *n* | 17 | 19 | 17 | 19 |
|  | Mean (SD) | 116 (38.7) | 96 (21.7) | –1 (22.8) | –11 (12.9) |
|  | *p*-value *vs.* week 0 | | | 0.917 | *p* < 0.01 |
|  | *p*-value 2 g *vs.* 4 g | | | 0.164 | |
| Fasting HDL-C (mg/dL) | | | | | |
| Week 0 | *n* | 18 | 19 |  |  |
|  | Mean (SD) | 49 (9.6) | 47 (9.7) |  |  |
| Week 8 | *n* | 17 | 19 | 17 | 19 |
|  | Mean (SD) | 51 (12.9) | 48 (11.7) | 2 (6.3) | 1 (4.8) |
|  | *p*-value *vs.* week 0 | | | 0.277 | 0.638 |
|  | *p*-value 2 g *vs.* 4 g | | | 0.681 | |
| 4-h postprandial HDL-C (mg/dL) | | | | | |
| Week 0 | *n* | 18 | 19 |  |  |
|  | Mean (SD) | 47 (9.4) | 44 (9.1) |  |  |
| Week 8 | *n* | 17 | 19 | 17 | 19 |
|  | Mean (SD) | 49 (13.4) | 45 (11.8) | 2 (6.4) | 1 (5.1) |
|  | *p*-value *vs.* week 0 | | | 0.325 | 0.454 |
|  | *p*-value 2 g *vs.* 4 g | | | 0.918 | |
| Fasting RLP-C (mg/dL) | | | | | |
| Week 0 | *n* | 18 | 19 |  |  |
|  | Mean (SD) | 8.4 (3.64) | 9.8 (4.13) |  |  |
| Week 8 | *n* | 17 | 19 | 17 | 19 |
|  | Mean (SD) | 6.9 (2.14) | 6.1 (3.18) | –1.8 (3.61) | –3.7 (2.82) |
|  | *p*-value *vs.* week 0 | | | 0.058 | *p <* 0.001 |
|  | *p*-value 2 g *vs.* 4 g | | | 0.206 | |
| 4-h postprandial RLP-C (mg/dL) | | | | | |
| Week 0 | *n* | 18 | 19 |  |  |
|  | Mean (SD) | 12.7 (5.20) | 13.6 (4.47) |  |  |
| Week 8 | *n* | 17 | 19 | 17 | 19 |
|  | Mean (SD) | 10.8 (3.11) | 8.4 (3.82) | –2.4 (4.81) | –5.2 (3.17) |
|  | *p*-value *vs.* week 0 | | | 0.055 | *p <* 0.001 |
|  | *p*-value 2 g *vs.* 4 g | | | *p <* 0.05 | |
| Fasting apolipoprotein B-48 (μg/mL) | | | | | |
| Week 0 | *n* | 18 | 19 |  |  |
|  | Mean (SD) | 4.5 (3.36) | 5.7 (3.18) |  |  |
| Week 8 | *n* | 17 | 19 | 17 | 19 |
|  | Mean (SD) | 3.7 (1.75) | 3.6 (1.80) | –0.6 (2.38) | –2.2 (2.58) |
|  | *p*-value *vs.* week 0 | | | 0.333 | *p <* 0.01 |
|  | *p*-value 2 g *vs.* 4 g | | | 0.149 | |
| 4-h postprandial apolipoprotein B-48 (μg/mL) | | | | | |
| Week 0 | *n* | 18 | 19 |  |  |
|  | Mean (SD) | 10.2 (5.91) | 11.0 (3.65) |  |  |
| Week 8 | *n* | 17 | 19 | 17 | 19 |
|  | Mean (SD) | 10.0 (4.34) | 8.6 (2.97) | –0.5 (3.93) | –2.4 (3.31) |
|  | *p*-value *vs.* week 0 | | | 0.603 | *p <* 0.01 |
|  | *p*-value 2 g *vs.* 4 g | | | 0.090 | |
| Fasting CRP (mg/dL) | | | | | |
| Week 0 | *n* | 18 | 19 |  |  |
|  | Mean (SD) | 0.29 (0.321) | 0.15 (0.083) |  |  |
| Week 8 | *n* | 17 | 19 | 17 | 19 |
|  | Mean (SD) | 0.31 (0.446) | 0.22 (0.179) | 0.04 (0.377) | 0.07 (0.145) |
|  | *p*-value *vs.* week 0 | | | 0.676 | *p <* 0.05 |
|  | *p*-value 2 g *vs.* 4 g | | | 0.932 | |
| Fasting urinary 8-epi-PGF2α (pg/mL) | | | | | |
| Week 0 | *n* | 18 | 19 |  |  |
|  | Mean (SD) | 393 (227.0) | 236 (161.6) |  |  |
| Week 8 | *n* | 17 | 19 | 17 | 19 |
|  | Mean (SD) | 415 (382.7) | 253 (154.6) | 9 (314.2) | 17 (100.0) |
|  | *p*-value *vs.* week 0 | | | 0.905 | 0.474 |
|  | *p*-value 2 g *vs.* 4 g | | | 0.886 | |

*CRP* C-reactive protein, *FAS* full analysis set, *h* hour, *HDL-C* high-density lipoprotein-cholesterol, *LDL-C* low-density lipoprotein-cholesterol, *Omega-3* omega-3 fatty acid ethyl esters, *RLP-C* remnant-like particle-cholesterol, *SD* standard deviation

**Supplementary Table S3** Overview of AEs by system organ class and preferred term (SAS)

|  | **Omega-3** | |
| --- | --- | --- |
|  | **2-g group** | **4-g group** |
|  | ***n* = 18** | ***n* = 19** |
| *AEs, n (%)* | *4 (22.2)* | *2 (10.5)* |
| Gastrointestinal disorders | 2 (11.1) | 0 (0.0) |
| Constipation | 1 (5.6) | 0 (0.0) |
| Diarrhea | 1 (5.6) | 0 (0.0) |
| Vomiting | 1 (5.6) | 0 (0.0) |
| Infections and infestations | 2 (11.1) | 1 (5.3) |
| Pharyngitis | 0 (0.0) | 1 (5.3) |
| Upper respiratory tract infection | 1 (5.6) | 0 (0.0) |
| Viral upper respiratory tract infection | 1 (5.6) | 0 (0.0) |
| Musculoskeletal and connective tissue disorders | 0 (0.0) | 1 (5.3) |
| Lumbar spinal stenosis | 0 (0.0) | 1 (5.3) |

*AEs* adverse events, *Omega-3* omega-3 fatty acid ethyl esters, *SAS* safety analysis set

**Supplementary Table S4** Measurement item list for biomarker evaluation

| **Fasting** | 4-h postprandial | |
| --- | --- | --- |
| Panel of lipids | | Panel of lipids |
| Phospholipids ([glycerophospholipid](http://www.weblio.jp/content/glycerophospholipid) [phosphatidylcholine, phosphatidylethanolamine, phosphatidylglycerol, phosphatidylserine, etc.], sphingophospholipids [sphingomyelin, etc.]), sphingolipid (ceramide, ganglioside, sulfatides, etc.), neutral lipids (monoacylglycerol, diacylglycerol, triacylglycerol, cholesteryl esters, dolichol, etc.), fatty acids (free fatty acids), acylcarnitine, bile acids, ubiquinone, and molecular species related to these biosynthesis and metabolism | | Phospholipids ([glycerophospholipid](http://www.weblio.jp/content/glycerophospholipid)  [phosphatidylcholine, phosphatidylethanolamine, phosphatidylglycerol, phosphatidylserine, etc.], sphingophospholipids [sphingomyelin, etc.]), sphingolipid (ceramide, ganglioside, sulfatides, etc.), neutral lipids (monoacylglycerol, diacylglycerol, triacylglycerol, cholesteryl esters, dolichol, etc.), fatty acids (free fatty acids), acylcarnitine, bile acids, ubiquinone, and molecular species related to these biosynthesis and metabolism |
| Panel of unsaturated fatty acid metabolites | |  |
| EPA metabolites (18-HEPE, 5-HEPE, Resolvin E1), DHA metabolites (17-HDHA [17-HdoHE], 7-HDHA [7-HdoHE], Resolvin D1, Resolvin D2, Resolvin D3, Maresin 1), AA metabolites (PGE2, PGD2, PGF2α, TXB2, 6-keto-PGF1α, LTB4) | |  |
| Panel of blood proteins | |  |
| PAI-1, TFPI, TNF-α, IL-1, IL-6, IL-8, IL-10, INFγ, MCP-1, ICAM-1, VCAM-1, eSelectin, SAA, PTX3, FABP4, leptin, adiponectin | |  |

*5-HEPE* 5-hydroxy eicosapentaenoate, *6-keto-PGF1α* 6-keto prostaglandin F1α, *7-HDHA (7-HdoHE)* 7-hydroxy docosahexaenoic acid, *17-HDHA (17-HdoHE)* 17-hydroxy docosahexaenoic acid, *18-HEPE* 18-hydroxy eicosapentaenoic acid, *FABP4* fatty acid binding protein 4, *IL-1* interleukin-1, *IL* interleukin, *INFγ* interferon gamma, *ICAM-1* intercellular adhesion molecule-1, *LTB4* leukotriene B4, *MCP-1* monocyte chemoattractant protein-1, *PAI-1* plasminogen activator inhibitor-1, *PG* prostaglandin, *PTX3* pentraxin 3, *SAA* serum amyloid A, *TFPI* tissue factor pathway inhibitor, *TNF-α* tumor necrosis factor alpha, *TXB2* thromboxane B2, *VCAM-1* vascular cell adhesion molecule-1

**Supplementary Table S5** (i) Coefficient of correlations with %FMD in panel of blood proteins and unsaturated fatty acid metabolites by treatment group. (ii) Spearman's rank correlation coefficient between change from week 0 on %FMD (fasting) and change from week 0 on panel of lipids (fasting) by treatment group. (iii) Spearman's rank correlation coefficient between percent change from week 0 on %FMD (fasting) and percent change from week 0 on panel of lipids (fasting) by treatment group

**(i)**

|  | **Omega-3** | |
| --- | --- | --- |
|  | **2-g group** | **4-g group** |
|  | ***n* = 18** | ***n* = 19** |
| **Coefficient of correlation** | **%FMD (fasting)**  **(8 week)** | **%FMD (fasting) (8 week)** |
| Panel of blood proteins (fasting) (8 week) | | |
| PAI1/SerpinE1 | 0.299 | –0.293 |
| TFPI | ­­–0.267 | 0.223 |
| IL-8/CXCL8 | –0.126 | –0.198 |
| MCP-1/CCL2 | 0.134 | 0.068 |
| ICAM1 | 0.186 | –0.014 |
| VCAM1 | –0.233 | 0.195 |
| e-Selectin | 0.134 | 0.227 |
| Serum amyloid A | –0.118 | 0.400 |
| Pentraxin 3/TSG-14 | 0.139 | –0.252 |
| FABP4 | 0.272 | –0.029 |
| Leptin | 0.170 | 0.011 |
| Adiponectin/Acrp30 | –0.315 | 0.155 |
| Panel of unsaturated fatty acid metabolites (fasting) (8 week) | | |
| 18-HEPE | –0.043 | 0.227 |
| 5-HEPE | 0.054 | 0.054 |
| 17-HDHA | 0.347 | –0.061 |
| PGE2 | –0.044 | 0.099 |
| TXB2 | –0.127 | –0.197 |

*5-HEPE* 5-hydroxy eicosapentaenoate, *17-HDHA (17-HdoHE)* 17-hydroxy docosahexaenoic acid, *18-HEPE* 18-hydroxy eicosapentaenoic acid, *CCL2* C-C motif chemokine ligand 2, *CXCL8* C-X-C motif chemokine ligand 8, *FMD* flow-mediated dilation, *IL-8* interleukin-8, *FABP4* fatty acid binding protein 4, *ICAM-1* intercellular adhesion molecule 1, *MCP-1* monocyte chemoattractant protein-1, *Omega-3* omega-3 fatty acid ethyl esters, *PAI-1* plasminogen activator inhibitor-1, *PGE2* prostaglandin E2, *TFPI* tissue factor pathway inhibitor, *TXB2* thromboxane B2, *VCAM-1* vascular cell adhesion protein 1

**(ii)** **[Uploaded as “Supplementary Table 5 ii & 5 iii.xlsx”]**

(X:Y): X indicates the number of carbons, Y indicates the number of double bond between carbons

1. X:Y): a- indicates that there is one acyl group attached to glycerol

(aa- X:Y): aa- indicates that there are two acyl group attached to glycerol

(e-X:Y): e- indicates that alkyl group attached to glycerol

(ae-X:Y): ae- indicates that acyl group and alkyl group attached to glycerol

(X:Y-OH): -OH indicated modification by hydroxyl group

*CE* cholesteryl esters, *Cer* ceramide, *Cer-1P* ceramide-1-phosphate, *DG* diacylglycerol, *DHSM* dihydrosphingomyelin, *FFA* free fatty acid, *GA1* GA1 ganglioside, *GM3* GM3 ganglioside, *Hex-Cer* Glc/Gal-ceramide, *Hex2-Cer* lactosylceramide, *Hex3-Cer* globotriaosylceramide, *LPC* lysophosphatidylcholine, *Omega-3* omega-3 fatty acid ethyl esters, *PC* phosphatidylcholine, *PE* phosphatidylethanolamine, *PI* phosphatidylinositol, *PS* phosphatidylserine, *phytocer* phytoceramide, *SM* sphingomyelin, *TG* triacylglycerol

**(iii) [Uploaded as “Supplementary Table 5 ii & 5 iii.xlsx”]**

(X:Y): X indicates the number of carbons, Y indicates the number of double bond between carbons

1. X:Y): a- indicates that there is one acyl group attached to glycerol

(aa- X:Y): aa- indicates that there are two acyl group attached to glycerol

(e-X:Y): e- indicates that alkyl group attached to glycerol

(ae-X:Y): ae- indicates that acyl group and alkyl group attached to glycerol

(X:Y-OH): -OH indicated modification by hydroxyl group

*CE* cholesteryl esters, *Cer* ceramide, *Cer-1P* ceramide-1-phosphate, *DG* diacylglycerol, *DHSM* dihydrosphingomyelin, *FFA* free fatty acid, *GA1* GA1 ganglioside, *GM3* GM3 ganglioside, *Hex-Cer* Glc/Gal-ceramide, *Hex2-Cer* lactosylceramide, *Hex3-Cer* globotriaosylceramide, *LPC* lysophosphatidylcholine, *Omega-3* omega-3 fatty acid ethyl esters, *PC* phosphatidylcholine, *PE* phosphatidylethanolamine, *PI* phosphatidylinositol, *PS* phosphatidylserine, *phytocer* phytoceramide, *SM* sphingomyelin, *TG* triacylglycerol

Supplementary Fig. S1 Study design


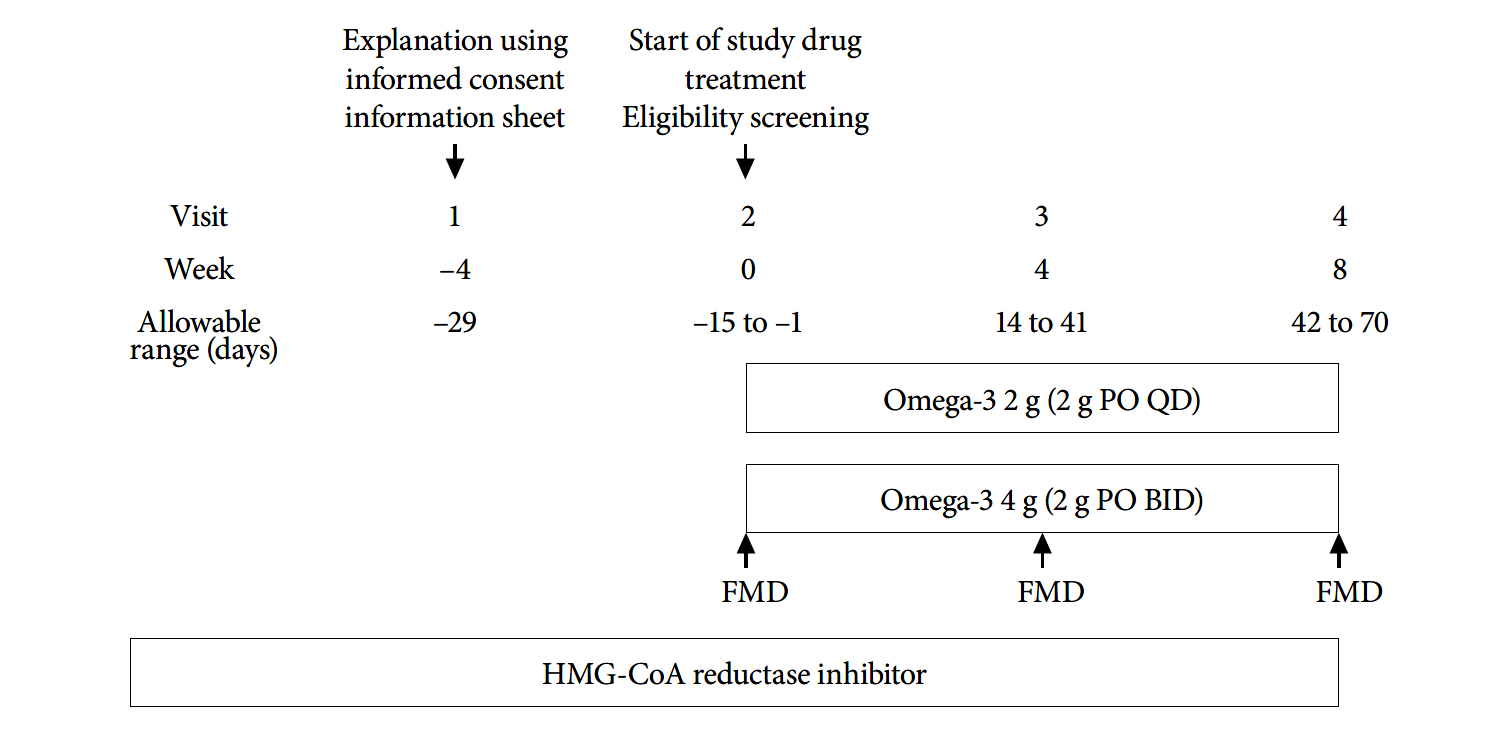


*BID* twice daily, *HMG-CoA* 3-hydroxy-3-methyl-glutaryl-coenzyme A, *FMD* flow-mediated dilation, *Omega-3* omega-3 fatty acid ethyl esters, *PO* oral, *QD* once daily

Supplementary Fig. S2 Patient disposition


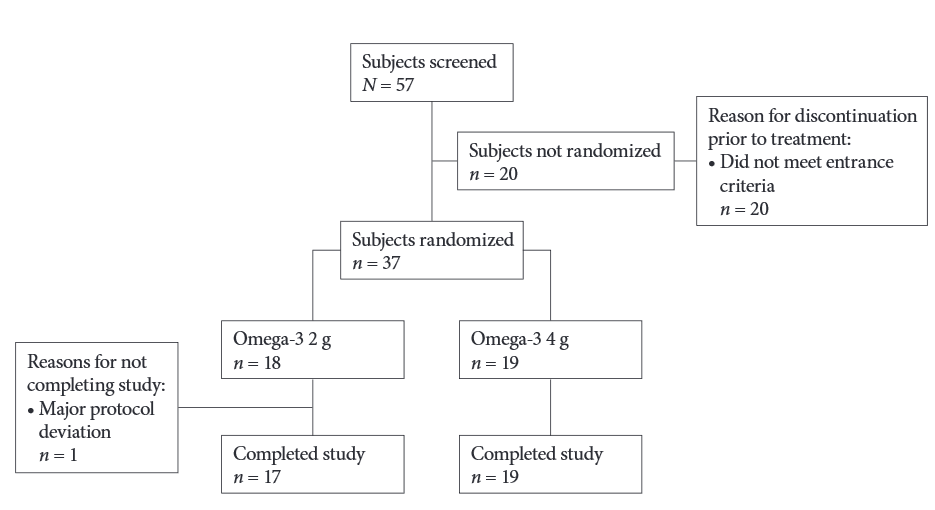

*Omega-3* omega-3 fatty acid ethyl esters

Supplementary Fig. S3 Mean change of 4-h postprandial %FMD from fasting %FMD in the Omega-3 2-g group and 4-g group


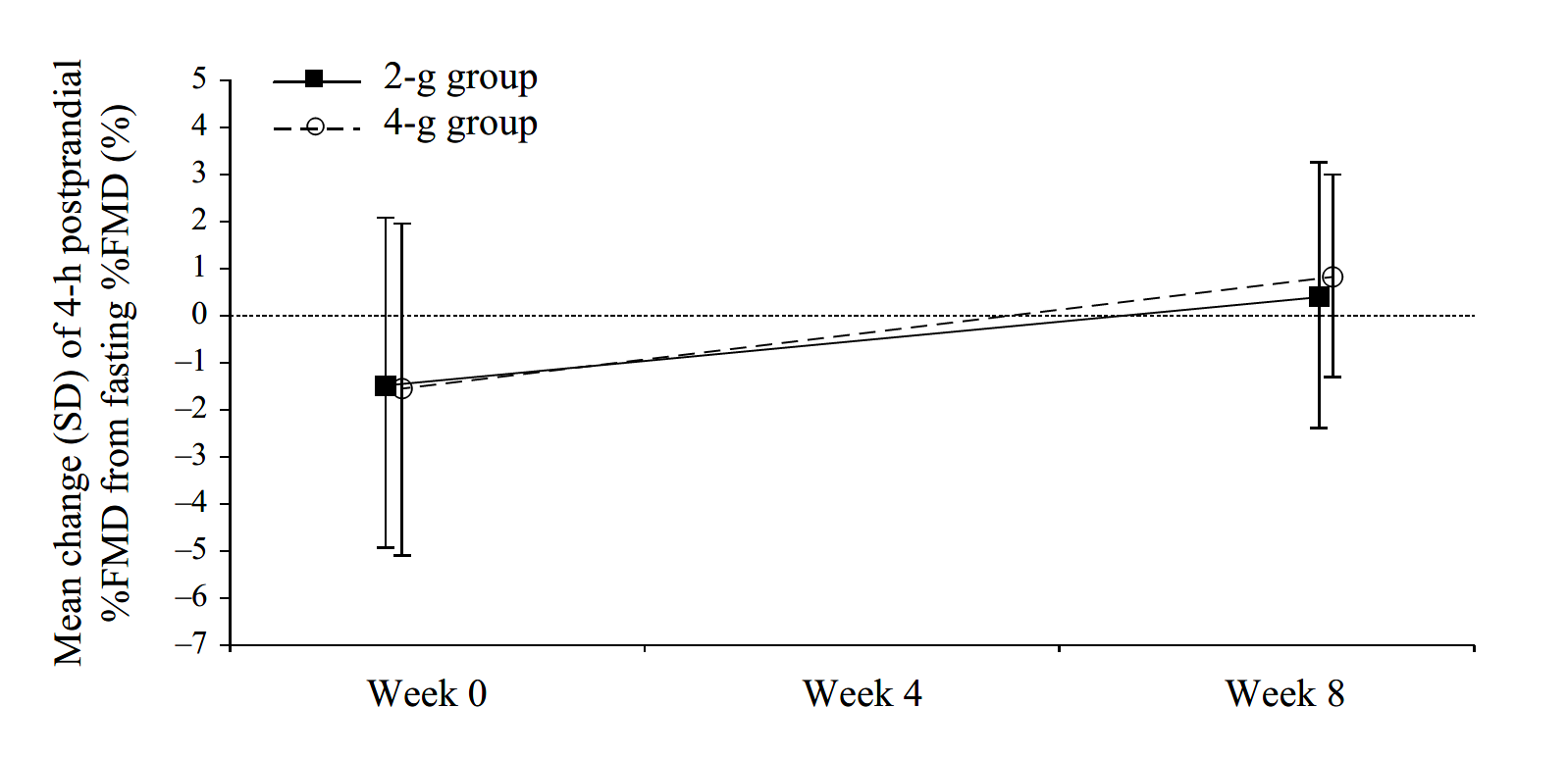


*FMD* flow-mediated dilation, *h* hour *Omega-3* omega-3 fatty acid ethyl esters, *SD* standard deviation
