# Supplementary material for: Discrepancy Between Fasting Flow-Mediated Dilation and Parameter of Lipids in Blood: A Randomized Exploratory Study of the Effect of Omega-3 Fatty Acid Ethyl Esters on Vascular Endothelial Function in Patients With Hyperlipidemia
Source: Adv Ther. 2020 Mar 21;37(5):2169–83. doi: 10.1007/s12325-020-01286-1 (PMC7467499; doi:10.1007/s12325-020-01286-1)
Supplement: Supplementary file 2 — Supplementary material 2 (PDF 79 kb) [file 12325_2020_1286_MOESM2_ESM.pdf]

Table S5 ii. Spearman's rank correlation coefficient between change from week 0 on %FMD (fasting) and change from week 0 (

	2 g group N = 18	4 g group N = 19
Spearman's Rank Correlation Coefficient	%FMD (fasting) (week 8)	%FMD (fasting) (week 8)
Panel of lipids (fasting) (8 week)		
Acylcarnitine(C10:0)	0.369	0.613
Acylcarnitine(C12:0)	0.377	0.556
Acylcarnitine(C12:1)	0.451	0.418
Acylcarnitine(C13:0)	0.560	0.441
Acylcarnitine(C13:1)	0.530	0.336
Acylcarnitine(C14:0)	0.449	0.472
Acylcarnitine(C14:1)	0.387	0.391
Acylcarnitine(C14:2)	0.343	0.316
Acylcarnitine(C16:0)	0.391	0.483
Acylcarnitine(C16:1)	0.376	0.456
Acylcarnitine(C18:0)	0.304	0.347
Acylcarnitine(C18:1)	0.246	0.443
Acylcarnitine(C18:2)	0.099	0.499
CE(a-18:2)	-0.265	-0.499
CE(a-20:4)	-0.215	-0.556
CE(a-20:5)	0.215	-0.275
CE(a-22:5)	-0.016	-0.368
CE(a-22:6)	0.116	-0.384
Cer(24:0)	-0.236	0.082
Cer-1P(24:0)	-0.077	0.002
Cer-1P(24:1)	0.077	0.244
DG(aa-34:1)	0.020	0.597
DG(aa-34:2)	0.004	0.547
DG(aa-34:3)	-0.059	0.447
DG(aa-35:1)	0.179	0.576
DG(aa-35:2)	0.188	0.293
DG(aa-36:0)	0.022	0.424
DG(aa-36:1)	0.061	0.617
DG(aa-36:2)	0.131	0.615
DG(aa-36:3)	0.059	0.495
DG(aa-36:4)	-0.029	0.470
DG(aa-36:5)	-0.174	0.487
DG(aa-38:2)	0.167	0.657
DG(aa-38:3)	0.123	0.611
DG(aa-38:4)	0.181	0.590
DG(aa-38:5)	0.093	0.550
DG(aa-38:6)	-0.041	0.456
DG(aa-38:7)	-0.015	0.502
DG(aa-40:5)	0.190	0.474
DG(aa-40:6)	0.066	0.570
DG(aa-40:7)	0.004	0.565
DG(aa-40:8)	-0.216	0.538
DHSM(16:0)	0.329	0.513
DHSM(18:0)	0.283	0.445
DHSM(20:0)	0.066	0.320
DHSM(22:0)	0.016	0.282
DHSM(23:0)	0.317	0.466
FFA(C10:0)	0.074	0.207
FFA(C12:0)	0.073	0.230
FFA(C12:1)	0.131	0.202
FFA(C14:0)	-0.072	0.236
FFA(C15:0)	-0.013	0.551
FFA(C15:1)	0.198	0.445
FFA(C18:0)	-0.218	0.084
FFA(C18:1)	0.191	0.286
FFA(C22:0)	-0.156	0.077
FFA(C22:1)	0.038	0.090
FFA(C24:0)	-0.246	0.292
FFA(C26:0)	0.175	-0.029
GA1(16:0)	0.349	0.540
GA1(16:1)	0.216	0.537
GM3(20:0)	0.100	0.036
GM3(22:1)	0.193	0.043

Table S5 ii. Spearman's rank correlation coefficient between change from week 0 on %FMD (fasting) and change from week 0 [illegible]

	2 g group *N* = 18	4 g group *N* = 19
Spearman's Rank Correlation Coefficient	%FMD (fasting) (week 8)	%FMD (fasting) (week 8)
GM3(23:0)	0.118	-0.098
GM3(23:1)	0.125	0.200
GM3(24:0)	-0.029	-0.098
GM3(24:1)	0.095	-0.148
GM3(24:2)	0.150	0.004
Hex-Cer(24:0)	-0.168	0.207
Hex2-Cer(24:1)	0.079	0.086
Hex3-Cer(16:0)	-0.175	0.080
Hex3-Cer(24:0)	0.248	-0.002
Hex3-Cer(24:1)	-0.181	0.045
LPC(a-16:1)	0.166	0.266
LPC(a-17:1)	0.537	0.406
LPC(a-18:3)	-0.258	0.155
LPC(a-20:0)	-0.133	0.288
LPC(a-20:1)	0.138	0.518
LPC(a-20:4)	0.122	0.418
LPC(a-20:5)	0.472	0.433
LPC(a-22:1)	0.321	0.168
LPC(a-22:4)	0.100	0.145
LPC(a-22:5)	0.168	0.304
LPC(a-22:6)	0.415	0.820
LPC(a-24:0)	0.099	0.423
LPC(e-18:1)	0.216	0.349
LPC(e-24:0)	0.183	0.235
LPC(e-24:1)	0.336	0.431
PC(aa-30:0)	0.091	0.307
PC(aa-30:1)	0.150	0.266
PC(aa-32:0)	0.261	0.214
PC(aa-32:1)	0.039	0.116
PC(aa-32:2)	-0.038	0.198
PC(aa-33:0)	0.295	0.490
PC(aa-33:2)	-0.104	0.365
PC(aa-34:0)	0.145	0.055
PC(aa-34:1)	0.093	0.361
PC(aa-34:2)	-0.136	0.332
PC(aa-34:3)	-0.116	0.120
PC(aa-34:4)	0.047	0.370
PC(aa-34:5)	0.259	0.651
PC(aa-35:0)	0.531	0.132
PC(aa-35:1)	0.424	0.368
PC(aa-35:2)	0.107	0.220
PC(aa-35:3)	0.191	0.324
PC(aa-35:4)	-0.016	0.329
PC(aa-35:5)	0.401	0.534
PC(aa-36:0)	0.196	0.071
PC(aa-36:1)	0.424	0.315
PC(aa-36:2)	0.036	0.586
PC(aa-36:3)	-0.127	0.175
PC(aa-36:4)	-0.222	0.406
PC(aa-36:5)	-0.419	0.204
PC(aa-37:1)	0.386	0.554
PC(aa-37:2)	0.147	0.417
PC(aa-38:1)	0.356	0.245
PC(aa-38:2)	0.177	0.424
PC(aa-38:3)	0.263	0.179
PC(aa-38:4)	0.420	0.297
PC(aa-38:5)	0.544	0.338
PC(aa-38:6)	0.639	0.549
PC(aa-38:7)	0.424	0.620
PC(aa-39:4)	0.508	0.130
PC(aa-39:5)	0.506	0.152
PC(aa-39:6)	0.665	0.175
PC(aa-40:4)	0.134	0.043
PC(aa-40:6)	0.472	0.676

Table S5 ii. Spearman's rank correlation coefficient between change from week 0 on %FMD (fasting) and change from week 0 c

	2 g group<br>N = 18	4 g group<br>N = 19
Spearman's Rank Correlation Coefficient	%FMD (fasting) (week 8)	%FMD (fasting) (week 8)
PC(aa-40:7)	0.487	0.559
PC(aa-42:5)	0.127	0.389
PC(aa-42:8)	-0.297	0.077
PC(aa-44:7)	0.343	0.365
PC(aa-44:8)	0.234	0.488
PC(aa-44:9)	0.038	-0.068
PC(ae-32:0)	0.229	0.390
PC(ae-32:1)	0.093	0.368
PC(ae-34:0)	0.061	0.160
PC(ae-34:5)	-0.049	0.050
PC(ae-36:1)	0.129	0.341
PC(ae-36:2)	0.045	0.188
PC(ae-36:3)	0.084	0.379
PC(ae-36:4)	0.258	0.227
PC(ae-36:5)	0.387	0.393
PC(ae-36:6)	0.057	0.138
PC(ae-38:2)	-0.134	0.243
PC(ae-38:3)	-0.057	0.267
PC(ae-38:4)	-0.048	0.363
PC(ae-38:6)	0.465	0.624
PC(ae-40:1)	0.130	0.470
PC(ae-40:2)	0.042	0.116
PC(ae-40:5)	0.258	0.648
PC(ae-40:7)	0.687	0.477
PC(ae-42:10)	0.581	0.402
PC(ae-42:2)	0.351	0.481
PC(ae-42:4)	0.063	0.509
PC(ae-42:5)	0.306	0.695
PC(ae-42:6)	0.187	0.300
PC(ae-42:7)	0.129	0.425
PC(ae-42:8)	0.079	0.379
PC(ae-42:9)	0.136	0.064
PC(ae-44:5)	0.161	0.563
PC(ae-44:6)	0.218	0.288
PC(ae-44:7)	0.254	0.374
PC(ae-44:8)	0.184	0.329
PC(ae-44:9)	0.043	0.440
PE(ae-36:3)	-0.168	0.175
PE(ae-38:4)	0.056	0.032
PE(ae-40:5)	-0.245	-0.107
PE(ae-40:6)	-0.222	0.025
PE(ae-40:7)	-0.100	0.104
PE(ae-42:6)	-0.243	-0.259
PE(ae-42:7)	-0.221	0.013
PI(aa-38:3)	0.025	0.018
PI(aa-40:6)	0.195	0.070
PS(aa-36:1)	0.098	0.054
PS(aa-36:2)	-0.070	0.161
PS(aa-38:5)	0.256	0.102
PS(aa-40:4)	0.000	0.029
PS(aa-40:5)	0.020	0.104
Phytocer(24:0)	-0.195	0.261
Phytocer(25:0)	-0.219	0.154
Phytocer(26:0)	0.246	0.032
SM(13:0)	0.445	0.797
SM(14:0)	0.220	0.558
SM(14:0-OH)	0.356	0.485
SM(14:1)	0.367	0.742
SM(15:0)	0.274	0.695
SM(15:1)	0.270	0.739
SM(16:0)	-0.029	0.427
SM(16:0-OH)	0.444	0.290
SM(17:0)	0.302	0.518
SM(18:0)	0.478	0.681

Table S5 ii. Spearman's rank correlation coefficient between change from week 0 on %FMD (fasting) and change from week 0 (

	2 g group<br>*N* = 18	4 g group<br>*N* = 19
Spearman's Rank Correlation Coefficient	%FMD (fasting) (week 8)	%FMD (fasting) (week 8)
SM(18:1)	0.524	0.702
SM(19:0)	0.279	0.488
SM(19:1)	0.256	0.191
SM(20:0)	-0.047	0.631
SM(20:1)	0.216	0.624
SM(20:2)	0.306	0.390
SM(21:0)	0.059	0.581
SM(21:1)	0.168	0.668
SM(22:0)	-0.050	0.334
SM(22:1)	0.191	0.468
SM(22:2)	0.374	0.609
SM(23:0)	0.184	0.572
SM(23:1)	0.322	0.668
SM(23:2)	0.546	0.638
SM(24:0)	0.064	0.416
SM(24:0-OH)	0.437	0.195
SM(24:1)	0.317	0.677
SM(24:1-OH)	0.586	0.617
SM(24:2)	0.363	0.622
SM(24:3)	0.503	0.431
SM(25:0)	0.102	0.472
SM(25:1)	0.240	0.549
SM(25:2)	0.157	0.497
SM(26:0)	-0.015	0.295
SM(26:1)	0.356	0.581
TG(aaa-49:1)	0.140	0.277
TG(aaa-49:2)	0.113	0.213
TG(aaa-54:0)	-0.327	-0.038
TG(aaa-56:0)	-0.060	-0.369

Table S5 iii. Spearman's rank correlation coefficient between percent change from week 0 on %FMD (fasting) and percent change frc

	2 g group<br>N = 18	4 g group<br>N = 19
Spearman's Rank Correlation Coefficient	%FMD (fasting) (week 8)	%FMD (fasting) (week 8)
Panel of lipids (fasting) (8 week)		
Acylcarnitine(C10:0)	0.418	0.657
Acylcarnitine(C12:0)	0.482	0.575
Acylcarnitine(C12:1)	0.575	0.539
Acylcarnitine(C13:0)	0.564	0.425
Acylcarnitine(C13:1)	0.368	0.440
Acylcarnitine(C14:0)	0.529	0.518
Acylcarnitine(C14:1)	0.400	0.554
Acylcarnitine(C14:2)	0.329	0.493
Acylcarnitine(C16:0)	0.461	0.446
Acylcarnitine(C16:1)	0.463	0.539
Acylcarnitine(C18:0)	0.396	0.366
Acylcarnitine(C18:1)	0.357	0.607
Acylcarnitine(C18:2)	0.307	0.621
CE(a-18:2)	-0.229	-0.407
CE(a-20:4)	-0.143	-0.507
CE(a-20:5)	0.286	-0.336
CE(a-22:5)	-0.264	-0.271
CE(a-22:6)	-0.014	-0.346
Cer(24:0)	-0.136	-0.061
Cer-1P(24:0)	-0.130	-0.111
Cer-1P(24:1)	0.218	0.139
DG(aa-34:1)	0.082	0.400
DG(aa-34:2)	0.089	0.286
DG(aa-34:3)	-0.036	0.307
DG(aa-35:1)	0.289	0.411
DG(aa-35:2)	0.293	0.239
DG(aa-36:0)	0.164	0.275
DG(aa-36:1)	0.229	0.511
DG(aa-36:2)	0.157	0.471
DG(aa-36:3)	0.061	0.371
DG(aa-36:4)	-0.032	0.418
DG(aa-36:5)	-0.139	0.339
DG(aa-38:2)	0.293	0.571
DG(aa-38:3)	0.154	0.432
DG(aa-38:4)	0.225	0.404
DG(aa-38:5)	0.111	0.296
DG(aa-38:6)	0.086	0.439
DG(aa-38:7)	0.118	0.346
DG(aa-40:5)	0.279	0.263
DG(aa-40:6)	0.189	0.414
DG(aa-40:7)	0.150	0.407
DG(aa-40:8)	0.021	0.229
DHSM(16:0)	0.282	0.461
DHSM(18:0)	0.300	0.511
DHSM(20:0)	0.100	0.314
DHSM(22:0)	0.064	0.221
DHSM(23:0)	0.364	0.436
FFA(C10:0)	0.143	0.127
FFA(C12:0)	0.161	0.136
FFA(C12:1)	0.173	0.182
FFA(C14:0)	0.029	0.079
FFA(C15:0)	0.025	0.371
FFA(C15:1)	0.216	0.247
FFA(C18:0)	-0.261	-0.150
FFA(C18:1)	0.364	0.143
FFA(C22:0)	-0.207	0.000
FFA(C22:1)	0.071	0.036
FFA(C24:0)	-0.139	0.250
FFA(C26:0)	-0.011	0.043
GA1(16:0)	0.436	0.582
GA1(16:1)	0.304	0.536
GM3(20:0)	0.014	-0.057
GM3(22:1)	0.143	-0.107

Table S5 iii. Spearman's rank correlation coefficient between percent change from week 0 on %FMD (fasting) and percent change fro

	2 g group N = 18	4 g group N = 19
Spearman's Rank Correlation Coefficient	%FMD (fasting) (week 8)	%FMD (fasting) (week 8)
GM3(23:0)	0.039	-0.179
GM3(23:1)	-0.138	0.218
GM3(24:0)	-0.043	-0.221
GM3(24:1)	0.050	-0.204
GM3(24:2)	0.243	0.014
Hex-Cer(24:0)	-0.014	0.011
Hex2-Cer(24:1)	0.209	0.054
Hex3-Cer(16:0)	-0.050	-0.061
Hex3-Cer(24:0)	0.035	0.200
Hex3-Cer(24:1)	-0.244	0.114
LPC(a-16:1)	0.304	0.404
LPC(a-17:1)	0.611	0.411
LPC(a-18:3)	-0.136	0.314
LPC(a-20:0)	0.000	0.300
LPC(a-20:1)	0.375	0.564
LPC(a-20:4)	0.232	0.425
LPC(a-20:5)	0.543	0.521
LPC(a-22:1)	0.367	0.161
LPC(a-22:4)	0.177	0.118
LPC(a-22:5)	0.232	0.254
LPC(a-22:6)	0.457	0.621
LPC(a-24:0)	0.104	0.311
LPC(e-18:1)	0.382	0.443
LPC(e-24:0)	0.193	0.407
LPC(e-24:1)	0.525	0.500
PC(aa-30:0)	0.039	0.300
PC(aa-30:1)	0.121	0.300
PC(aa-32:0)	0.254	0.396
PC(aa-32:1)	0.168	0.039
PC(aa-32:2)	0.150	0.286
PC(aa-33:0)	0.339	0.450
PC(aa-33:2)	0.100	0.532
PC(aa-34:0)	0.171	0.211
PC(aa-34:1)	0.182	0.346
PC(aa-34:2)	-0.036	0.339
PC(aa-34:3)	-0.011	0.282
PC(aa-34:4)	0.175	0.446
PC(aa-34:5)	0.450	0.557
PC(aa-35:0)	0.579	0.154
PC(aa-35:1)	0.561	0.468
PC(aa-35:2)	0.243	0.521
PC(aa-35:3)	0.300	0.593
PC(aa-35:4)	0.146	0.257
PC(aa-35:5)	0.632	0.550
PC(aa-36:0)	0.207	0.089
PC(aa-36:1)	0.396	0.454
PC(aa-36:2)	0.171	0.604
PC(aa-36:3)	-0.036	0.225
PC(aa-36:4)	-0.107	0.432
PC(aa-36:5)	-0.229	0.332
PC(aa-37:1)	0.371	0.629
PC(aa-37:2)	0.282	0.596
PC(aa-38:1)	0.379	0.350
PC(aa-38:2)	0.214	0.375
PC(aa-38:3)	0.289	0.143
PC(aa-38:4)	0.607	0.321
PC(aa-38:5)	0.561	0.368
PC(aa-38:6)	0.586	0.389
PC(aa-38:7)	0.525	0.350
PC(aa-39:4)	0.529	0.168
PC(aa-39:5)	0.425	0.325
PC(aa-39:6)	0.704	0.282
PC(aa-40:4)	0.164	0.061
PC(aa-40:6)	0.429	0.650

Table S5 iii. Spearman's rank correlation coefficient between percent change from week 0 on %FMD (fasting) and percent change fro

	2 g group N = 18	4 g group N = 19
Spearman's Rank Correlation Coefficient	%FMD (fasting) (week 8)	%FMD (fasting) (week 8)
PC(aa-40:7)	0.579	0.457
PC(aa-42:5)	0.175	0.529
PC(aa-42:8)	-0.479	0.043
PC(aa-44:7)	0.404	0.436
PC(aa-44:8)	0.211	0.311
PC(aa-44:9)	-0.050	-0.196
PC(ae-32:0)	0.396	0.461
PC(ae-32:1)	0.189	0.514
PC(ae-34:0)	0.125	0.143
PC(ae-34:5)	-0.064	0.143
PC(ae-36:1)	0.321	0.396
PC(ae-36:2)	0.250	0.400
PC(ae-36:3)	0.189	0.311
PC(ae-36:4)	0.343	0.139
PC(ae-36:5)	0.500	0.507
PC(ae-36:6)	-0.021	0.000
PC(ae-38:2)	0.114	0.225
PC(ae-38:3)	0.125	0.225
PC(ae-38:4)	0.096	0.318
PC(ae-38:6)	0.425	0.468
PC(ae-40:1)	0.139	0.396
PC(ae-40:2)	0.089	0.214
PC(ae-40:5)	0.321	0.704
PC(ae-40:7)	0.686	0.346
PC(ae-42:10)	0.543	0.171
PC(ae-42:2)	0.396	0.532
PC(ae-42:4)	0.236	0.414
PC(ae-42:5)	0.439	0.750
PC(ae-42:6)	0.371	0.461
PC(ae-42:7)	0.279	0.536
PC(ae-42:8)	0.239	0.454
PC(ae-42:9)	0.336	0.036
PC(ae-44:5)	0.371	0.461
PC(ae-44:6)	0.350	0.468
PC(ae-44:7)	0.314	0.532
PC(ae-44:8)	0.264	0.454
PC(ae-44:9)	0.146	0.468
PE(ae-36:3)	-0.029	0.139
PE(ae-38:4)	0.143	0.039
PE(ae-40:5)	-0.231	0.025
PE(ae-40:6)	-0.161	0.254
PE(ae-40:7)	-0.071	0.039
PE(ae-42:6)	-0.161	-0.211
PE(ae-42:7)	-0.226	0.125
PI(aa-38:3)	0.021	-0.179
PI(aa-40:6)	0.207	0.089
PS(aa-36:1)	0.175	0.207
PS(aa-36:2)	0.089	0.264
PS(aa-38:5)	0.361	0.161
PS(aa-40:4)	-0.029	-0.020
PS(aa-40:5)	0.311	0.130
Phytocer(24:0)	-0.107	0.154
Phytocer(25:0)	-0.175	0.032
Phytocer(26:0)	0.275	-0.175
SM(13:0)	0.364	0.657
SM(14:0)	0.304	0.550
SM(14:0-OH)	0.107	0.407
SM(14:1)	0.364	0.704
SM(15:0)	0.354	0.682
SM(15:1)	0.257	0.689
SM(16:0)	0.150	0.332
SM(16:0-OH)	0.457	0.329
SM(17:0)	0.389	0.614
SM(18:0)	0.593	0.754

Table S5 iii. Spearman's rank correlation coefficient between percent change from week 0 on %FMD (fasting) and percent change fro

	2 g group<br>*N* = 18	4 g group<br>*N* = 19
Spearman's Rank Correlation Coefficient	%FMD (fasting) (week 8)	%FMD (fasting) (week 8)
SM(18:1)	0.636	0.686
SM(19:0)	0.289	0.586
SM(19:1)	0.332	0.155
SM(20:0)	0.104	0.525
SM(20:1)	0.246	0.718
SM(20:2)	0.332	0.489
SM(21:0)	0.107	0.475
SM(21:1)	0.275	0.779
SM(22:0)	0.071	0.325
SM(22:1)	0.261	0.550
SM(22:2)	0.429	0.511
SM(23:0)	0.186	0.514
SM(23:1)	0.371	0.696
SM(23:2)	0.554	0.757
SM(24:0)	0.150	0.304
SM(24:0-OH)	0.336	0.164
SM(24:1)	0.450	0.586
SM(24:1-OH)	0.564	0.639
SM(24:2)	0.514	0.704
SM(24:3)	0.521	0.607
SM(25:0)	0.154	0.475
SM(25:1)	0.432	0.600
SM(25:2)	0.400	0.707
SM(26:0)	0.064	0.318
SM(26:1)	0.436	0.571
TG(aaa-49:1)	0.146	-0.004
TG(aaa-49:2)	0.186	0.129
TG(aaa-54:0)	-0.207	-0.368
TG(aaa-56:0)	-0.175	-0.518
